# Overexpression of ORCA3 and G10H in *Catharanthus roseus* Plants Regulated Alkaloid Biosynthesis and Metabolism Revealed by NMR-Metabolomics

**DOI:** 10.1371/journal.pone.0043038

**Published:** 2012-08-20

**Authors:** Qifang Pan, Quan Wang, Fang Yuan, Shihai Xing, Jingya Zhao, Young Hae Choi, Robert Verpoorte, Yuesheng Tian, Guofeng Wang, Kexuan Tang

**Affiliations:** 1 Plant Biotechnology Research Center, SJTU-Cornell Institute of Sustainable Agriculture and Biotechnology, Fudan-SJTU-Nottingham Plant Biotechnology R&D Center, School of Agriculture and Biology, Shanghai Jiao Tong University, Shanghai, People’s Republic of China; 2 Natural Products Laboratory, Institute of Biology Leiden, Leiden University, Leiden, The Netherlands; Instituto Valenciano De Investigaciones Agrarias, Spain

## Abstract

In order to improve the production of the anticancer dimeric indole alkaloids in *Catharanthuse roseus*, much research has been dedicated to culturing cell lines, hairy roots, and efforts to elucidate the regulation of the monoterpenoid indole alkaloid (MIA) biosynthesis. In this study, the ORCA3 (Octadecanoid-derivative Responsive *Catharanthus* AP2-domain) gene alone or integrated with the G10H (geraniol 10-hydroxylase) gene were first introduced into *C. roseus* plants. Transgenic *C. roseus* plants overexpressing ORCA3 alone (OR lines), or co-overexpressing G10H and ORCA3 (GO lines) were obtained by genetic modification. ORCA3 overexpression induced an increase of AS, TDC, STR and D4H transcripts but did not affect CRMYC2 and G10H transcription. G10H transcripts showed a significant increase under G10H and ORCA3 co-overexpression. ORCA3 and G10H overexpression significantly increased the accumulation of strictosidine, vindoline, catharanthine and ajmalicine but had limited effects on anhydrovinblastine and vinblastine levels. NMR-based metabolomics confirmed the higher accumulation of monomeric indole alkaloids in OR and GO lines. Multivariate data analysis of ^1^H NMR spectra showed change of amino acid, organic acid, sugar and phenylpropanoid levels in both OR and GO lines compared to the controls. The result indicated that enhancement of MIA biosynthesis by ORCA3 and G10H overexpression might affect other metabolic pathways in the plant metabolism of *C. roseus*.

## Introduction

In the 1950s, the Canadian scientists Robert Noble and Charles Beer first isolated and characterized the alkaloid vinblastine from *Catharanthus roseus* leaves [1]. Currently, more than 130 monoterpenoid indole alkaloids (MIA) have been found in different parts of *C. roseus* [2]. Of the MIAs, dimeric indole (bisindole) alkaloids vincristine and vinblastine are being used clinically as anticancer agents. Despite their importance, sources of the compounds are still limited. *C. roseus* is the main source of MIAs but the low yields have been an obstacle to production of the compounds. The high medical value and extremely low yields from *C. roseus* [3] of vinblastine and vincristine have motivated extensive studies to elucidate MIA biosynthesis and regulation.

The biosynthesis of MIA begins with the coupling of a terpenoid (the iridoid glycoside secologanin) and an indole moiety (tryptamine). The terpenoid part starts from geraniol produced by the mevalonate-independent pathway (MEP). The indole part derives, via anthranilate, from chorismate which is a major branching point in the shikimate pathway. Chorismate is also the precursor of a wide range of phenolic compounds [4], among others, via prephenate. A series of genes encoding MIA biosynthetic enzymes (anthranilate synthase alpha subunit, Asα; 1-deoxy-D-xylulose synthase, DXS; tryptophan decarboxylase, TDC; strictosidine synthase, STR; strictosidine beta-glucosidase, SGD; geraniol 10-hydroxylase, G10H; desacetoxyvindoline 4-hydroxylase, DAT; apoplastic peroxidase, CrPrx; secologanin synthase, SLS) have been characterized, cloned and overexpressed alone or in combination in cell cultures and hairy roots of *C. roseus* in the past decade [5], [6], [7], [8], [9], [10], [11], [12], [13], [14], [15]. Transformation of transcription factors (ORCA2 and ORCA3) [16], transporters (ATP-binding cassette transporter, ABC) [17] and heterologous genes (Bcl-2 Associated X protein, Bax) [18] were also performed in *C. roseus* cells and hairy roos. Genetic modification is a good approach to study the regulation of MIA biosynthesis and to improve the production of targeted MIAs in *C. roseus*, thus lower the production costs for these very expensive clinical medicines.

**Figure 1:**
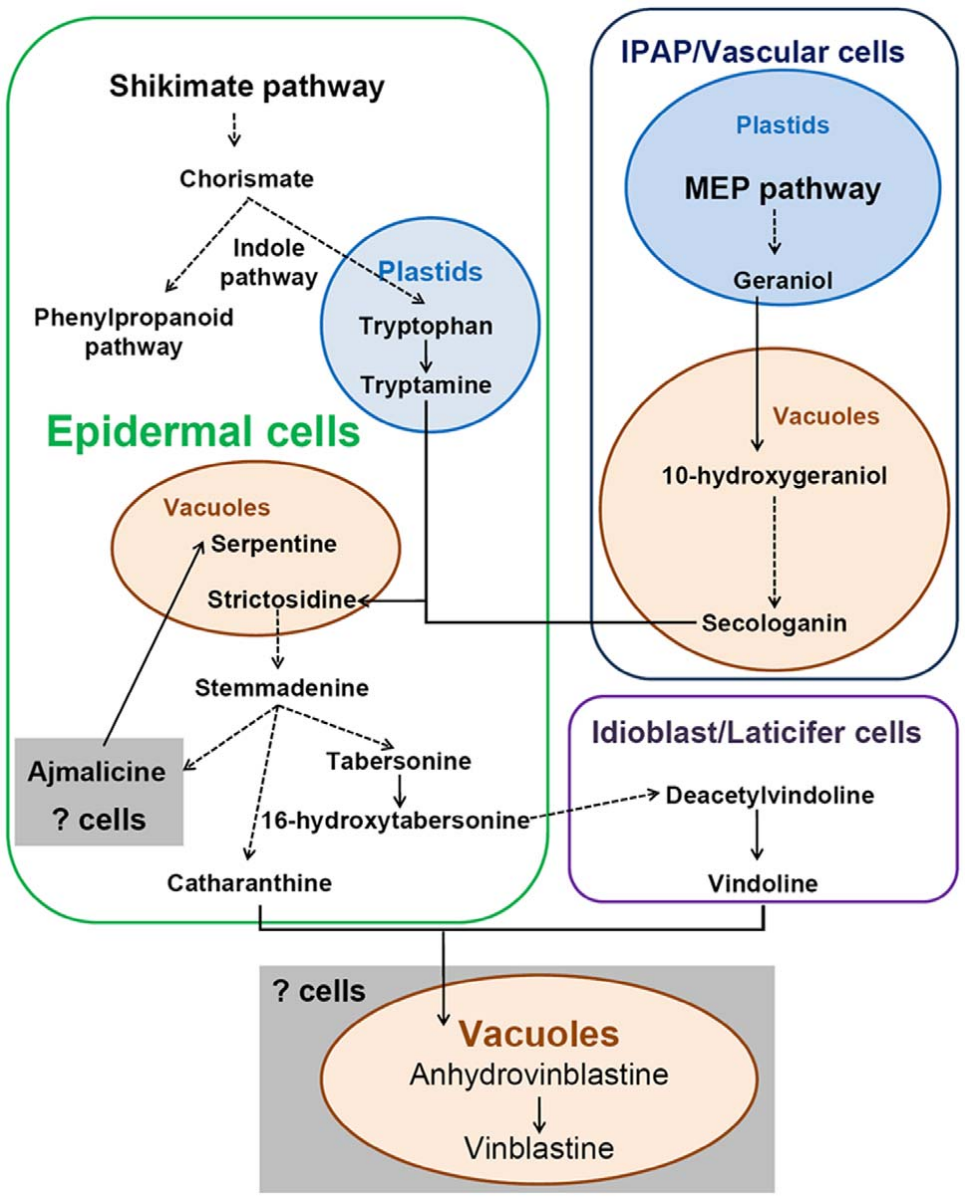
The location and brief biosynthesis pathway of indoles, iridoids and MIAs.

However, MIA production in in vitro cultures seems to be limited by the complexity of metabolic regulation, which involves intracellular compartmentation, different cell types and tissue differentiation in *C. roseus* (Fig. 1). The MEP pathway which produces iridoids is localized in plastids of the internal phloem associated parenchyma (IPAP) of young developing aerial organs of *C. roseus* [19]. The products of the indole pathway and some of the monomeric indole (monoindole) alkaloids accumulate in the epidermis of *C. roseus* aerial organs [20], [21], [22]. The bisindole alkaloids vinblastine and vincristine have been found to be produced and stored in vacuoles [23]. Monoindole alkaloid vindoline is one of the precursors of the dimers. Its last two biosynthetic steps take place in the specialized laticifer and idioblast cells of the green parts of plants [24]. Using cell suspension cultures and hairy roots is successful for the production of some of the monomers (ajmalicine and serpentine) but not for the production of vindoline [25], [26], [27]. There is no report about significant production of the bisindole alkaloids vinblastine and vincristine in cell cultures or hairy roots. Apparently different cell types in the leaf are needed for the biosynthesis of bisindole alkaloid precursors (Fig. 1). Neither cell cultures nor hairy roots covers all the leaf cell types. Therefore gene-transformation into plants is a promising alternative to improving MIA production.

ORCA3, as a jasmonate-inducible AP2/ERF-domain transcription factor, is a key regulator of several important genes in MIA biosynthesis, such as *DXS*, *AS*, *TDC*, *STR* and *D4H* [28]. *ORCA3* alone or combined with other genes has been transformed into *C. roseus* cell cultures or hairy roots to understand the MIA biosynthesis regulation [28], [29], [30], [31]. However, promoter analysis of *STR*, *TDC*, *G10H* and *DAT* complicated the regulatory landscape of MIA synthesis [32], [33], [34], [35]. Recently, a basic helix-loop-helix (BHLH) transcription factor CrMYC2 was reported as the major activator of JA-responsive ORCA gene expression [36]. The current work also discusses the transcriptional correlation between CRMYC2 and ORCA3 genes under ORCA3 overexpression.

The iridoid pathway is one of the precursor pathways for the MIA biosynthesis and usually regarded as the limiting step in cell and tissue cultures of *C. roseus* [37], [38]. With the aim of improving the MIA production of pharmacological interest, several points remain to be elucidated in this highly complex pathway in *C. roseus*. To the best knowledge, the starting point of the iridoid pathway is the enzyme, G10H, a cytochrome P450 monooxygenase that hydroxylates geraniol to form 10-hydroxy-geraniol. This step is not regulated by ORCA3 when tested in cell suspension cultures [28]. Previous reports indicate that overexpression of G10H in the hairy roots improved production of several MIAs when combined with the addition of precursors, hormones or under certain forms of stress [13], [35]. In the present work, G10H integrated with ORCA3 was co-overexpressed in *C. roseus* plants to investigate their effect on MIA biosynthesis without exogenous treatment.

NMR-based metabolomics is a powerful tool to uncover the metabolic status of complex plant matrices with a broad spectrum of both primary and secondary metabolites. NMR spectroscopy is a non-targeted method to provide a comprehensive fingerprint and profile of a wide range of metabolites based on signals generated from all proton-containing chemicals. Mass spectroscopy and NMR-based metabolomics have been employed in combination with multivariate data analysis to study plant physiology [39], [40], classification of different plants ecotypes and genotypes [41], [42], [43], plant metabolic network and its correlation with the genome (functional genomics). Moreover, NMR-based metabolomics has been applied to distinguish transgenic plants from non-transgenic plants and inspect the potential effect of transgenic events on plant metabolism for bio-safety evaluation [44], [45], [46], [47].

In the current work, we successfully inserted *ORCA3* and *G10H* into *C. roseus* plants to improve MIA accumulation. The changes of gene transcripts and alkaloid concentrations were analyzed to better understand the regulation of monoindole and bisindole alkaloid biosynthesis from a systemic view. NMR-based metabolomics was performed on these transgenic plants to investigate the effect of ORCA3 and G10H overexpression on the whole metabolome of *C. roseus* plants.

## Results

### Generation of G10H and ORCA3 Overexpressing Transgenic Catharanthus roseus Plants

**Figure 2:**
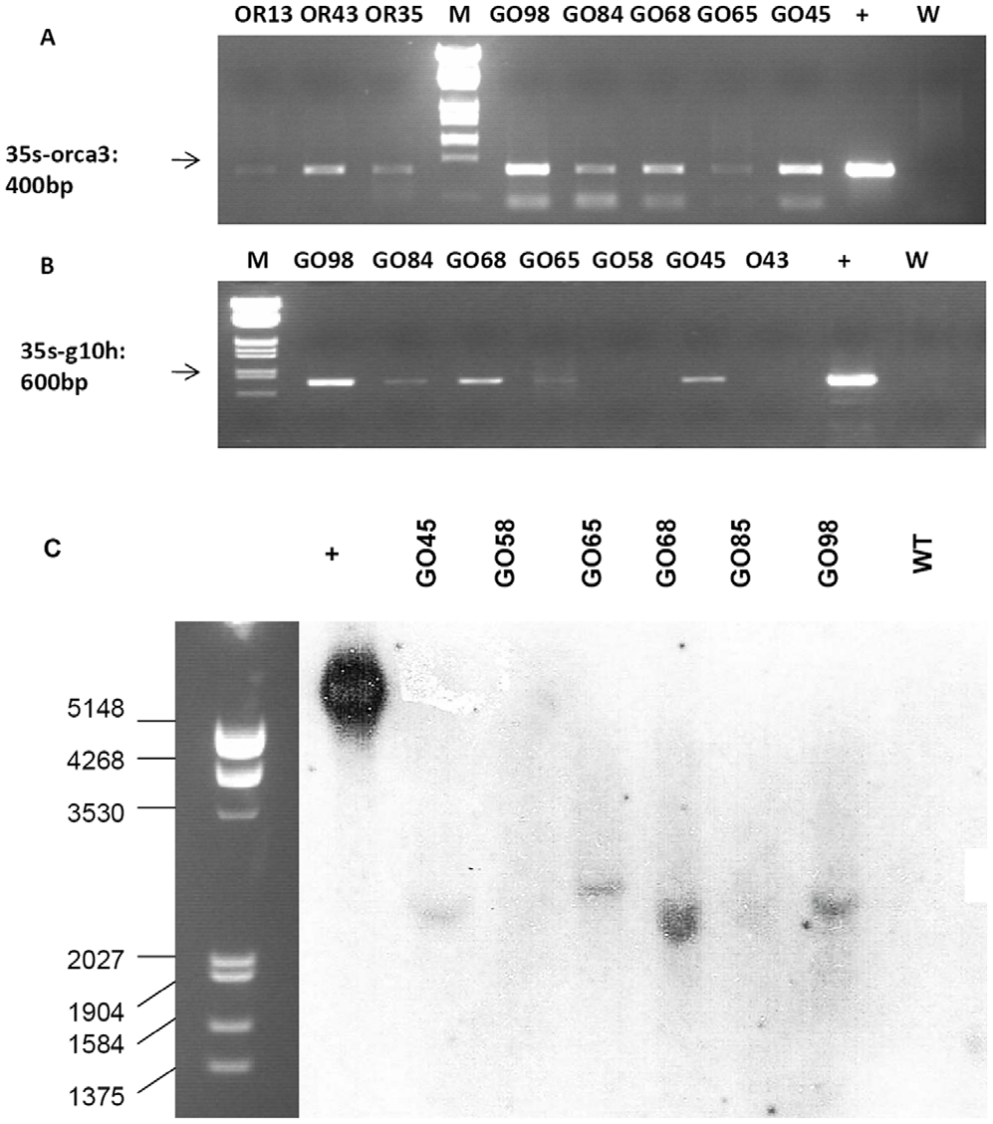
Representative PCR and Southern-blot analyses of independent transgenic *C. roseus* plants. A: PCR analysis for the presence of ORCA3 in OR and GO plants; +: Positive control (pGO plasmid); W: wild type plant (negative control). B: PCR analysis for the presence of G10H in OR and GO plants; +: Positive control; W: wild type plants (negative control). C: Southern-blot analysis of transgenic *C. roseus* plants; +: Positive control (GO plasmid digested by *Sal*I); WT: wild type plants.

The constructs of ORCA3 gene alone or integrated with G10H gene were introduced into *C. roseus* plants by *A. tumefaciens*-mediated transformation (Fig. S1). The hypocotyls from *C. roseus* seedlings were cut and incubated with *A. tumefaciens* for gene insertion. Callus was induced and selected by kanamycin. Shoots were regenerated from the surviving callus and selected by kanamycin continuously. After three rounds of kanamycin (90 mg L^−1^) selection, a total of 16 independent plantlets transformed with the construct of ORCA3 (OR) and 39 independent plantlets transformed with the construct of G10H and ORCA*3* (GO) survived. Three OR lines and five GO lines which showed a fast root growth in root initiation medium containing 90 mg L^−1^ kanamycin were chosen to transplant into the field for next analysis. Their transgenic status was confirmed by molecular analysis (PCR and Southern-blot) (Fig. 2 and Fig. S2). Fifteen control plants were grown side by side. All the plants were slender in the growth chamber but grew well after transplanted into the field (Fig. S3A–S3D). The growth of transgenic plants was not changed considerably compared to the controls (Fig. S3E and S3F).

### Transcription Level Analysis

To investigate the expression of the introduced genes and their effect on the transcript profile of alkaloid biosynthesis, besides ORCA3 and G10H, AS, TDC (indole pathway), DXS (MEP pathway), STR (MIA pathway), D4H (vindoline biosynthetic pathway) and CRMYC2 (JA signaling pathway) genes were also analyzed by real time PCR.

**Figure 3:**
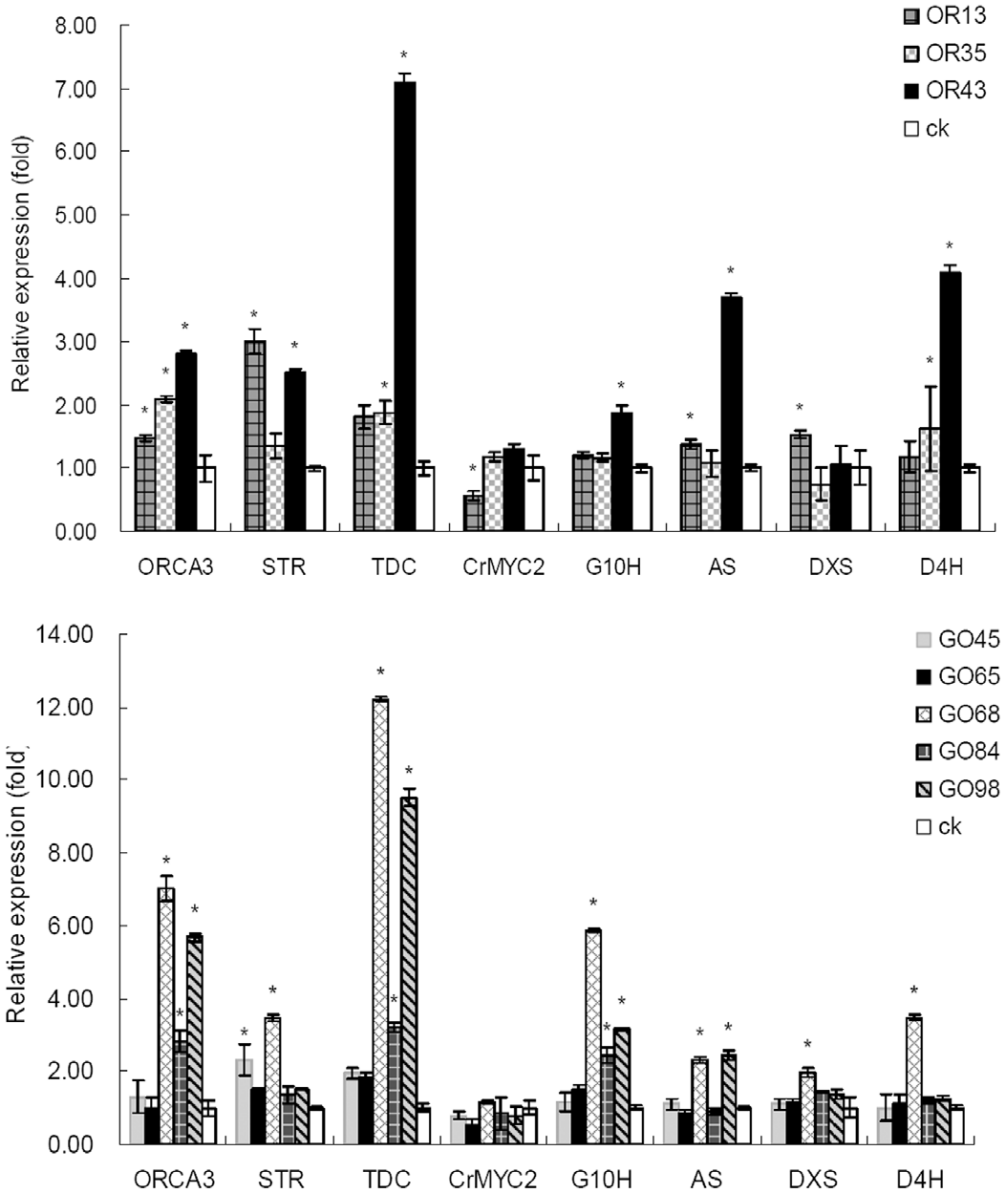
ORCA3, G10H, *tdc*, *str* and *crmyc2* transcripts in OR and GO plants. Error bars represent the standard deviation of triplicate runs for Real Time PCR. *: significant difference (*p*<0.05 by ANOVA).

ORCA3 transcripts showed a significant increase in all OR lines and four GO lines compared with the controls (Fig. 3). Three GO lines (GO68, GO84 and GO98) with highly enhanced ORCA3 transcripts also showed a significant increase of G10H transcripts. These results confirmed that G10H and ORCA3 were overexpressed in *C. roseus* plants. The variance of G10H and ORCA3 overexpression levels in different transgenic lines may result from different gene copy number and position effects which are common in *Agrobacterium*-transformed plants [48], [49]. The transcripts of G10H and ORCA3 are correlated to each other because they are in the same T-DNA insertion. Nonetheless G10H transcripts in OR lines as that in the controls was lower than that in GO lines, which indicated that ORCA3 overexpression didn’t induce G10H expression in OR lines.

The key indole genes *AS* and *TDC* transcripts were induced and paralleled ORCA3 expression level in OR and GO lines (Fig. 3). STR and D4H transcripts also responsed to ORCA3 overexpression, but showed corresponded less with the increased levels of ORCA3 transcripts, especially in GO lines. DXS transcripts showed less of relationship with ORCA3 overexpression in both OR and GO lines. The results implied that ORCA3 had an up-regulation effect on the genes in the indole and MIA pathways in *C. roseus* plant. The expression level of CRMYC2 showed no significant difference among OR, GO and control lines.

### Effect on the Monoterpenoid Indole Alkaloid Pathway in Catharanthus roseus Plants

Investigation by HPLC was carried out to study the effect of ORCA3 and G10H overexpression on the accumulation of monoindole alkaloids (vindoline, catharanthine and ajmalicine) and bisindole alkaloids (anhydrovinblastine and vinblastine) in *C. roseus* leaves (Fig. S4).

All OR and GO lines showed higher levels of monoindole alkaloids vindoline, catharanthine and ajmalicine than the control. The vindoline accumulation ranged from 0.78–2.83 mg g^−1^ DW in OR lines and from 1.25–3.00 mg g^−1^ DW in GO lines when the average in control lines was 0.70 mg g^−1^ DW. The catharanthine accumulation was variable with the range of 3.65–5.79 mg g^−1^ DW in OR lines and 3.78–5.36 mg g^−1^ DW in GO lines when the average in control lines was 1.99 mg g^−1^ DW. Ajmalicine was accumulated with the range of 0.07–0.10 mg g^−1^ DW in OR lines and 0.14–0.49 mg g^−1^ DW in GO lines, while the average in control lines was 0.05 mg g^−1^ DW. However, only two OR lines and two GO lines showed an increase of bisindole alkaloids anhydrovinblastine and vinblastine. The change of vinblastine accumulation was inconsistent with that of anhydrovinblastine accumlation. Line OR43 had the highest level of anhydrovinblastine and vinblastine, at 10.25 mg g^−1^ DW with 1.08-fold up and at 0.27 mg g^−1^ DW with 10.19-fold up compared with the controls, respectively. The results implied that ORCA3 and G10H overexpression had a stronger influence on the biosynthesis of monoindole alkaloids than that of bisindole alkaloids. The accumulation of vinblastine was more predictable of the level of anhydrovinblastine than that of their precursors vindoline and catharanthine.

**Figure 4:**
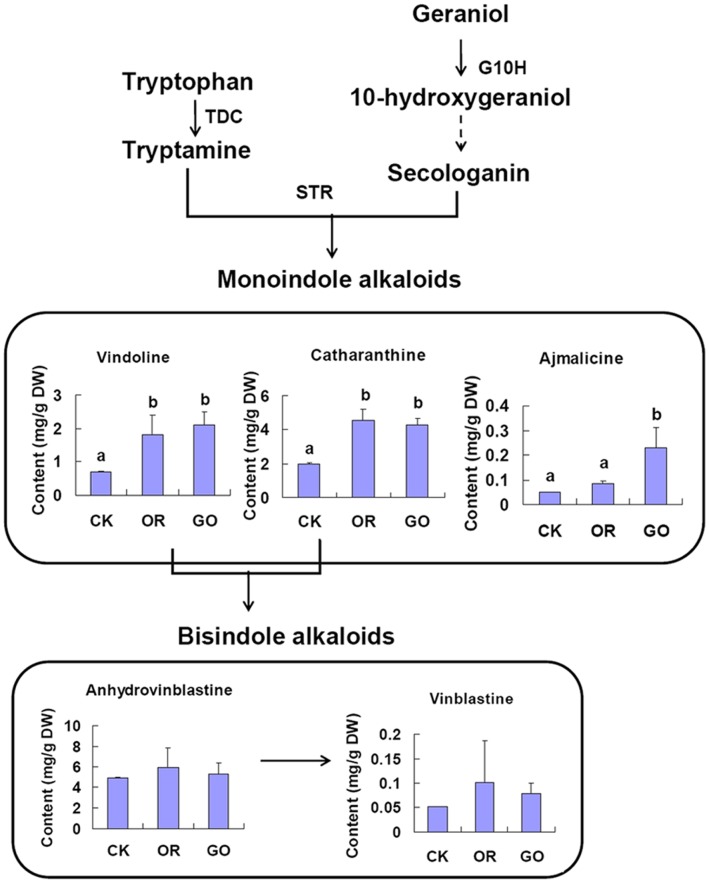
The average levels of several important MIAs in OR, GO and control lines. The value of CK is the average of 15 control plants. Different letters indicate significant difference (*p*<0.05 by ANOVA). The value of OR is the average of pooled samples from 3 OR lines; the value of GO is the average of pooled samples from 5 GO lines.

In order to analyse the functional effect between overexpression of ORCA3 and ORCA3 with G10H co-overexpression on the MIA parthway, a one-way analysis of variance (ANOVA) was conducted to detect the difference of average levels of monoindole and bisindole alkaloids among OR, GO and control lines (Fig. 4). There was no significant difference of vindoline and catharanthine contents between OR and GO lines, and both were significantly higher compared to the controls. GO lines showed a significantly higher level of ajmalicine than OR and control lines. The contents of bisindole alkaloids anhydrovinblastine and vinblastine exhibited no significant difference among OR, GO and control lines. These results indicated that overexpression of *ORCA3* combined with a constitute gene *G10H* had a greater influence on the biosynthesis of monoindole alkaloids than *ORCA3* alone overexpression, especially on ajmalicine accumulation. Both co-overexpression and overexpression of only ORCA3 had limited effect on the accumulation of bisindole alkaloids.

### Effect of G10H and ORCA3 Overexpression on Catharanthus roseus Plant Metabolism

**Figure 5:**
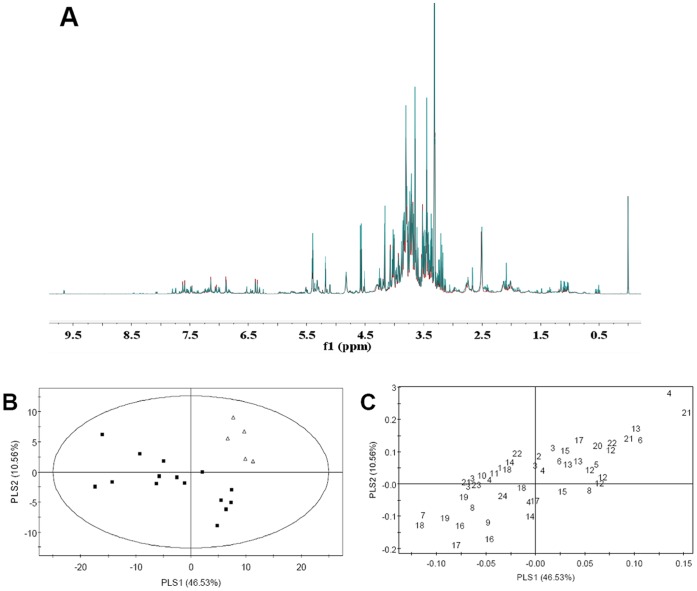
^1^H-NMR investigation and multivariate data analysis of GO and control lines. A: ^1^H-NMR spectra of GO (in green) and control (in red) lines. B: Score plot of PLS-DA of GO (Δ) and control (▪) lines. C: Loading plot of PLS-DA. The numbers represent metabolites following table 1.

Both OR and GO lines were used and studied by NMR-based metabolomics for the potential influence of G10H and ORCA3 overexpression on *C. roseus* plant metabolism. Around 40 primary and secondary metabolites were elucidated using a combination of 2D-NMR (*J*-resolved and COSY) and referred to the previous literature NMR profiling data [39], [43], [50] and the in-house database with more than 500 common metabolites (Table S1). In ^1^H-NMR spectrum of both GO and control lines, signals in the aliphatic area (δ 0.4 to δ 3.3) were assigned to a series of amino acids and organic acids, such as threonine, alanine, arginine, glutamic acid, glutamine, malic acid, citric acid and succinic acid. Another organic acid fumaric acid showed its signal at δ 6.56. Glucose and sucrose were two major sugars found in the NMR spectra (δ 3.3 to δ 5.5) of *C. roseus* plants. In the aromatic area (δ 6.0 to δ 9.0), phenolic compounds (2,3-DHBA, phenylpropanoids and flavonoids), terpenoids (secologanin) and MIAs (strictosidine, vindoline, catharanthine and serpentine) were identified. Secologanin shows a signal at δ 9.68 and vindoline has a characteristic signal at δ 0.52. There are two types of caffeoylquinic acids detected and identified in *C. roseus* leaves: chlorogenic acid (5-*O*-Caffeoyl quinic acid) and 4-*O*-Caffeoyl quinic acid. Their signals of H-2′, H-5′, H-6′, H-7′ and H-8′ always appear side by side and differ around 0.05 ppm from each other [39]. ^1^H-NMR spectra of OR and GO lines were not observed any unpredictable signals compared with the controls, but signals of some metabolites showed difference in the peak integration (Fig. 5A).

Principal component analysis (PCA), an unsupervised clustering method, and supervised partial least squares-discriminate analysis (PLS-DA) were conducted to analyze the ^1^H-NMR data sets of all samples. PCA score plot identified the difference between the sample sets and showed a partial separation of GO and control lines on PC1 and PC6 (Fig. S5A). PLS-DA score plot displayed a clearer metabolic discrimination between GO and control lines on PLS1 and PLS2, and determined the variables (i.e., metabolite signals) responsible for the separation (Fig. 5B). The PLS-DA model was validated by the permutation method through 20 applications (Fig. S5B). GO lines were located at the positive sides of PLS1 and PLS2. The control plants were scattered in the rest of the score plot and showed clear differences between individual plants. Based on PLS-DA loading plot (Fig. 5C), signals of alanine, glutamic acid, arginine, glucose, sucrose, 2,3-butanediol, quercetin-3-*O*-glucoside, strictosidine, vindoline and catharanthine were present at higher levels in GO plants, while the controls showed higher levels of threonine, secologanin, 2,3-DHBA, chlorogenic acid, 4-*O*-Caffeoyl quinic acid, malic acid and fumaric acid.

**Figure 6:**
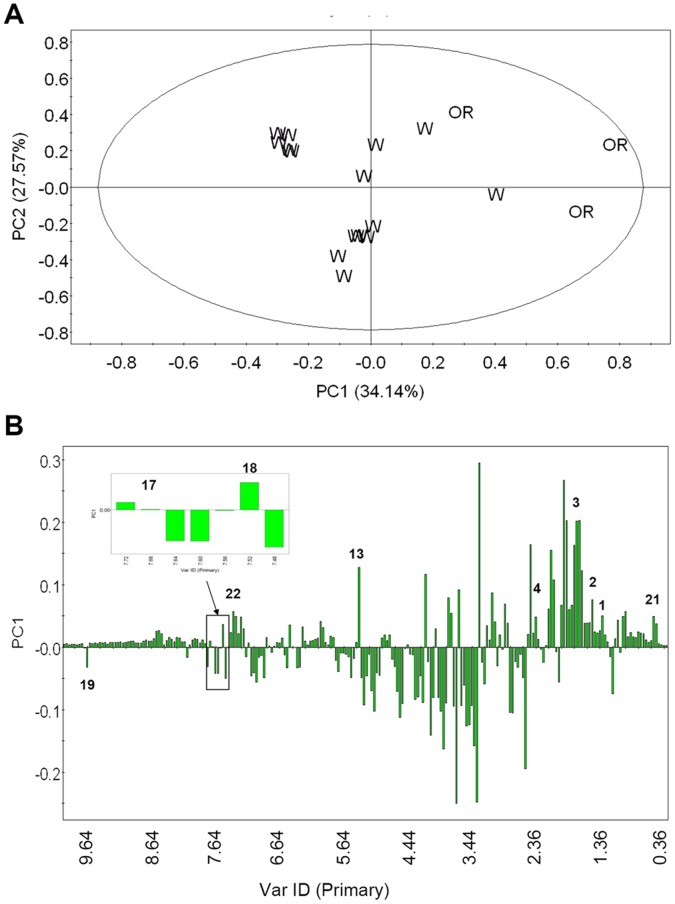
Multivariate data analysis of OR and control lines based on ^1^H-NMR spectra. A: PCA score plot of OR lines (OR) and control lines (W); B: PCA column plot of PC1. The numbers represent metabolites following table 1.

In PCA score plot, OR and control lines were mainly separated by PC1 (Fig. 6A). OR lines were all at the positive side of PC1 and showed individual difference within the group. The controls were mostly located at the negative side of PC1. Analyzed by the column loading plot of PC1, signals of amino acids, sucrose, MIAs, 2,3-DHBA and 4-*O*-Caffeoyl quinic acid showed higher contents while signals of organic acids, glucose, flavonoids and secologanine showed lower contents in the OR lines than in the controls (Fig. 6B).

According to the loadings plot, the metabolites responsible for the separation were selected to perform ANOVA to confirm their contribution to metabolic discrimination between overexpression lines and control plants (Table 1). The contents of alanine, strictosidine, vindoline and catharanthine were present significantly higher in both OR and GO lines than the controls. OR lines also contained significantly higher levels of threonine, arginine, glutamic acid, sucrose, 2,3-DHBA and 4-*O*-Caffeoyl quinic acid while GO lines had significant increases of 2,3-butanediol, glucose and quercetin-3-*O*-glucoside compared with control lines. Besides, OR lines showed significant decreases in the levels of malic acid and secologanine while GO lines showed slight decreases of these two compounds.

**Table 1:** ANOVA results for selected signals from OR, GO transgenic and wild type plants.

		OR lines		GO lines	
No	Metabolites	Relative intensity ratio	p value	Relative intensity ratio	p value
1	Threonine	4.69	0.000	1.21	0.352
2	Alanine	4.19	0.002	2.00	0.004
3	Arginine	4.80	0.001	1.23	0.210
4	Glutamic acid	2.49	0.012	1.19	0.259
5	2,3-butanediol	1.10	0.534	1.48	0.001
6	Glutamine	–	–	–	–
7	Ethanol	1.09	0.797	1.11	0.683
8	Succinic acid	–	–	–	–
9	malic acid	0.31	0.005	0.88	0.568
10	Quinic acid	–	–	–	–
11	Lactic acid	–	–	–	–
12	Glucose	0.40	0.055	1.83	0.017
13	Sucrose	2.11	0.001	1.38	0.081
14	Kaempferol	0.76	0.403	1.29	0.170
15	Quercetin-3-*O*-glucoside	0.69	0.262	1.57	0.022
16	Chlorogenic acid	0.74	0.172	0.97	0.577
17	4-*O*-Caffeoyl quinic acid	1.34	0.028	1.18	0.162
18	2,3-DHBA	1.60	0.048	0.96	0.781
19	Secologanin	0.23	0.008	0.70	0.146
20	Strictosidine	1.36	0.039	3.25	0.000
21	Vindoline	2.28	0.012	2.82	0.000
22	Catharanthine	1.63	0.003	1.46	0.003
23	Serpentine	1.06	0.276	2.00	0.553
24	Fumaric acid	0.33	0.344	0.87	0.808

* *p* value <0.05: significant difference.

* –: overlapped signal, not quantitatively analyzed.

## Discussion

As a key transcription factor in the JA-induced MIA pathway, ORCA3 overexpression resulted in the up-regulation of several alkaloid-related biosynthetic genes (such as *DXS*, *AS*, *TDC*, *STR* and *D4H*) in *C. roseus* cell cultures [28]. In this study, ORCA3 gene was overexpressed in *C. roseus* plants and activiated the expression of AS, TDC, STR and D4H but not DXS. When ORCA3 alone or integrated with G10H was overexpressed in *C. roseus* hairy roots, only STR and SLS expression were enhanced while there was no changes in neither TDC nor CPR transcripts and a decrease in SGD transcripts [29], [30]. Accumulation of G10H transcript was not enhanced in the OR lines, suggesting that ORCA3 overexpression didn’t regulate G10H expression in *C. roseus* plants. These results comfirmed the up-regulation role of ORCA3 in the MIA pathway in *C. roseus* plants as in its cell cultures. CRMYC2 gene is an immediate-early jasmonate-responsive gene which encodes the basic helix-loop-helix (bHLH) transcription factor CrMYC2 to regulate ORCA gene expression [36]. CrMYC2 binds to the qualitative sequence in the ORCA3 jasmonate-responsive element (JRE) in a transcriptional cascade to active the biosynthesis genes expression. Our research suggests that ORCA3 alone overexpression has no feedback-effect on CRMYC2 transcripts.

ORCA3 overexpression induced increased levels of several key genes transcripts in the MIA pathway, and consequently, enhanced the production of MIAs. Overexpressing ORCA3 alone or co-overexpressing ORCA3 and G10H in *C. roseus* plants strongly stimulated the accumulation of monomeric alkaloids but only slightly affected the accumulation of bisindole alkaloids anhydrovinblastine and vinblastine. The bisindole alkaloids are produced and stored in vacuoles of specific cells. Their monomeric precursors catharanthine and vindoline are made in other cells and cellular compartments [23]. When the increase of key genes transcripts resulted in the enhanced productivity of monomeric terpenoid indole alkaloids, the flux to bisindole alkaloids might be limited by compartmentation and intercellular transport. Another possibility is that the competition of monoindole alkaloids biosynthesis limited the flux to the production of bisindole alkaloids anhydrovinblastine and vinblastine. Anhydrovinblastine is produced by the dimerization reaction of vindoline and catharanthine in vacuoles, which is further converted into vinblastine [51]. The results in this study reveal that the accumulation of anhydrovinblastine was high and not affected by ORCA3 and G10H overexpression. Despite the presence of anhydrovinblastine, the production of vinblastine and vincristine was low. This indicates that in *C. roseus* the major bisindole alkaloid stored in vacuoles is anhydrovinblastine and there might be other factors involved in regulating the biosynthesis of vinblastine and vincristine.

In previous studies, overexpression of G10H and ORCA3 in *C. roseus* hairy roots improved catharanthine production but has no effect on other alkaloids [35]. When ORCA3 alone was overexpressed in the hairy roots, a decrease in several MIA (included catharanthine) was observed [13], [31]. Although an ORCA3-transformed *C. roseus* cell line can improve the accumulation of total alkaloids, deficiency to biosynthesize vindoline causes the failure to synthesize bisindole alkaloids in cultured cells [12]. Apparently the biosynthesis of MIAs requires a high level of tissue differentiation, multicellular organization, and compartmentation. Compared to cell and hairy root systems, a whole plant is a complete intact biological system with a high level of differentiation which contains many cell types and compartments required for the MIA biosynthesis (Fig. 1). When ORCA3 and G10H were introduced into *C. roseus* plants, overexpression had an influence not only on the accumulation of catharanthine and ajamalicine but also vindoline. Surprisingly, as revealed by NMR-based metabolomics, secologanin did not significantly accumulate in GO lines. In fact, its level in GO plants was lower than in the controls. G10H is an endoplasmic-reticulum (ER)-anchored enzyme starting the secoiridoid pathway by catalyzing the hydroxylation of geraniol to 10-hydroxygeraniol [52], [53]. It takes at least 7 steps to biosynthesize secologanin from 10-hydroxygeraniol [51], which might limit the effect of G10H overexpression on secologanin accumulation. In OR lines, the significant decrease of secologanine might result from the enhancement of MIA biosynthesis.

**Figure 7:**
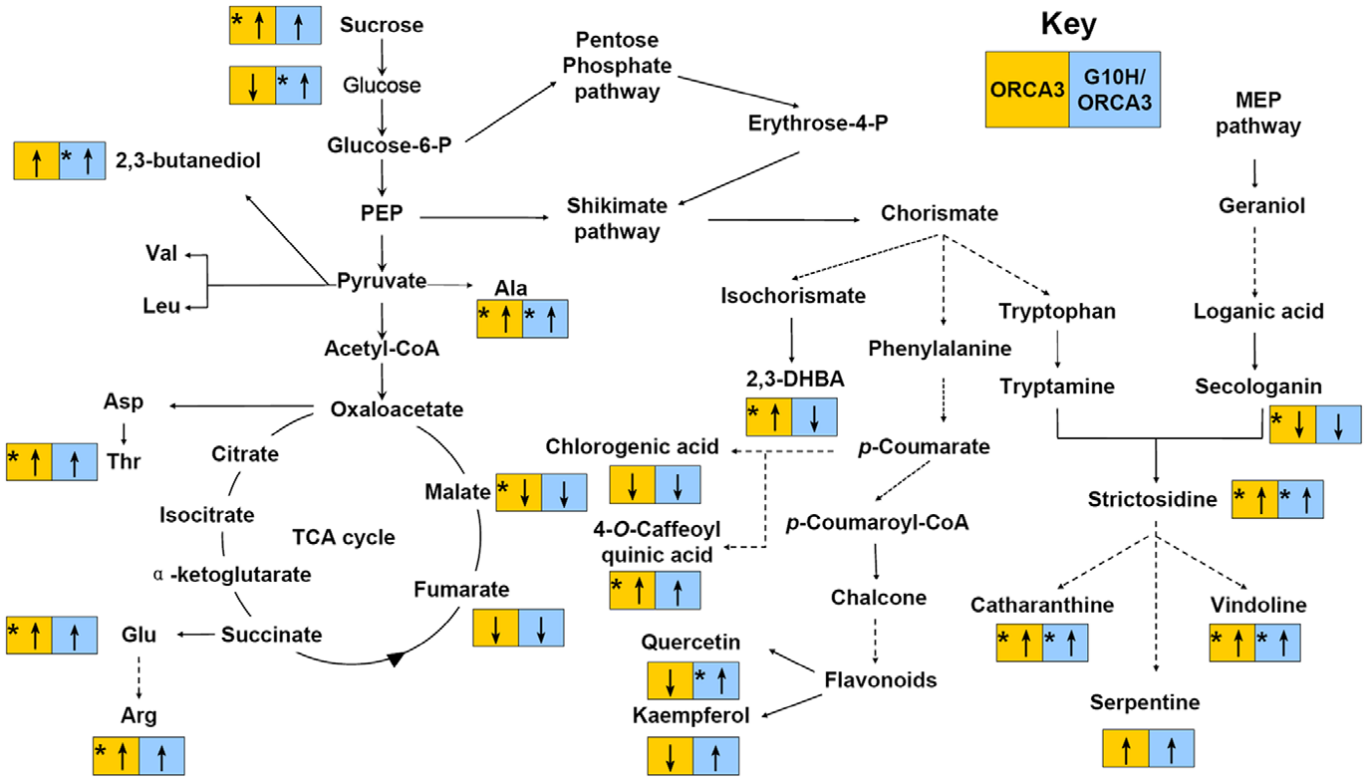
Schematic effects of ORCA3, or with G10H, overexpression on the metabolism of *C. roseus* plants. Orange box is for ORCA3 overexpression (the OR lines) and blue box is for G10H and ORCA3 co-overexpression (the GO lines). The up arrow in the box represents the increase of metabolite content. The down arrow in the box represents the decrease of metabolite content. Arrows with star in the box represent significant difference (*p*<0.05 by ANOVA) of metabolite content compared with the controls.

Integrated investigation of the metabolome in OR, GO and control lines indicated that both primary and secondary metabolism were modulated by G10H and ORCA3 overexpression (Fig. 7). Bialaphos resistant (BAR) gene overexpressing *Arabidopsis* plants also showed higher levels of alanine and threonine [47]. Transgenic tomato accumulates more glutamate and less sugar than non-transgenic ones [54]. Changes in levels of amino acids and sugars might be associated with the gene overexpression. As a transcriptional factor, ORCA3 regulates not only secondary but also primary metabolite biosynthetic genes involved in MIA biosynthesis [28]. Increase of a series of amino acid levels and decrease of organic acid levels in OR lines, as well as more accumulation of glucose, alanine, 2,3-butanediol in GO lines, indicated that primary metabolic fluxes might be affected by ORCA3 overexpression in *C. roseus* plants. Compared with GO lines, more changes of metabolite contents in OR lines implied that overexpression of a regulator gene might have a greater influence on plant metabolism than that of a structural gene.

Phenylpropanoids were also investigated in this study. Change occurred to the levels of 2,3-DHBA (the isochorismic pathway), Caffeoyl quinic acids (the phenylalanine pathway) and flavonoids (general phenylpropanoid pathway) among OR, GO and control lines. In OR lines, the levels of 2,3-DHBA and 4-*O*-Caffeoyl quinic acid significantly increased and the levels of chlorogenic acid, kaempferol and quercetin-3-*O*-glucoside slightly decreased. In GO lines the content of quercetin-3-*O*-glucoside significantly increased, 4-*O*-Caffeoyl quinic acid and kaempferol showed increases in their levels while chlorogenic acid and 2,3-DHBA showed slight decreases in their levels (Fig. 7). Both phenylpropanoids and MIAs derive from the intermediate chorismic acid in the shikimate pathway [4]. Vacuoles accumulate both MIA and phenylpropanoids like flavonoids. Flavonoids and alkaloids also co-accumulate in single epidermis cells of *C. roseus* leaves [21]. Previous study has reported that ectopic overexpression of tryptophan feedback-resistant anthranilate synthase holoenzyme (ASαβ) in *C. roseus* hairy roots increased the accumulation of phenolic compounds, especially flavonoids catechin [55]. Our results were inconsistent with the previous study. ORCA3 overexpression, or with G10H co-overexpression, in *C. roseus* plants constantly pushed the metabolic flux towards the indole pathway (tryptophan and tryptamine) via chorismate and had a feedback on the other branches from chorismate pathway towards phenylpropanoids. The corresponding change of phenylpropanoid levels suggested that metabolic flux control of branch-point pathway might be well coordinated among the branches from chorismate in the shikimate pathway.

The current work demonstrates that overexpression of a regulatory gene (*ORCA3*) and a structural gene (*G10H*) in the MIA pathway results in increases of monoterpenoid indole alkaloids (strictosidine, vindoline, catharanthine and ajmalicine) accumulation in *C. roseus* plants, and also has a feedback on phenylpropanoid biosynthesis. The result suggests that ORCA gene might thus have a broader effect on the cellular differentiation towards a phenotype of epidermal cells. Since ORCA3 overexpression did not have a strongly positive effect on the accumulation of bisindole alkaloids, the study points to a separation of regulation for this last step in the biosynthesis of these alkaloids, which does not occur in the epidermis but might occur in specialized idioblasts deep in *C. roseus* leaves. Regulating MIA biosynthesis to improve production requires a more rational engineering on the complex biosynthetic pathways involving different cell types.

## Materials and Methods

### Construction of Plasmid Containing ORCA3 and G10H

The 612 bp CDS of ORCA3 and 1479 bp CDS of G10H were cloned from *C. roseus* plant (Pacifica Cherry Red, Pan American Seed Co, IL, USA) by RT-PCR using RNA extracted from leaves of *C. roseus* seedlings with Plant Total RNA Isolation Kit (Watson Biotechnologies, Inc, Shanghai, China). In RT-PCR, ORCA3-F1 (5′-ACTAGTATGTCCGAAGAAATCAT-3′), ORCA3-R1 (5′-GGTCACCTTAATATCGTCTCTTCT-3′), G10H-F1 (5′-ACTAGTATGGATTACCTTACCAT-3′) and G10H-R1 (5′-GGTCACCAAGGGTGCTTGGTACAGC-3′) were used as the forward and reverse primers. The parameters for the standard PCR were as follows: 94°C, 2 min; 35 cycles: 94°C, 30 s; 54°C, 30 s, 72°C, 90 s; 72°C, 10 min. The two fragments were first ligated into plasmid pGEM-T easy for sequence confirmation, and then excised and cloned into a modified pMD18 EXP vector through *Spe*I and *Bst*EII sites, respectively, which resembled the genes with a cauliflower mosaic virus 35S (*CaMV 35S*) promoter and *Agrobacterium tumefaciens nos* terminator. Finally, two expression cassettes (p35s-ORCA3-nos and p35s-G10H-nos) were inserted into the *Sal*I and *Sma*I sites of the expression vector pCAMBIA2300 (p2300) to get the single gene-containing vector pOR (pCAMBIA2300::p35s-ORCA3-nos) and pG (pCAMBIA2300::p35s-G10H-nos). The expression cassette (p35s-ORCA3-nos) was inserted into pCAMBIA2300::p35s-G10H-nos to generate double gene-containing vector pGO (pCAMBIA2300::p35s-G10H-nos::p35s-ORCA3-nos). The vector p2300 contains a selection marker gene *NPTII* (neomycin phosphotransferase gene conferring resistance to kanamycin). The pCAMBIA2300::p35s-ORCA3-nos and pCAMBIA2300::p35s-G10H-nos::p35s-ORCA3-nos were then transferred into *Agrobacterium tumefaciens* strain EHA105 by a conventional freezing-and-melting method, and the resulting strains were used in the transformation of *C. roseus*.

### Transformation, Regeneration and Cultivation of Catharanthus roseus Plants

Seeds of *C. roseus* (Pacific Cherry Red cultivar) were purchased from PanAmerican Seed Company (U.S.A.). Seed surfaces were sterilized in 75% (v/v) ethanol for 2 min and 5% (v/v) NaClO for another 5 min, and then washed five times with sterile distilled water. Seeds were germinated on Petri plates containing MS (Murashige and Skoog 1962) basal medium. Cultures were grown under a 16 h light and 8 h dark photoperiod at 25±2°C. When seedlings reached 1–1.5 cm in length (5-day old), the hypocotyls, about 5 mm, were excised from the germinated seedlings and used as the explants in *A. tumefaciens*-mediated transformation. The explants were immerged in liquid MS medium with 100 µM acetosyringone in a steriled glass tissue culture tube. The tube was sonicated for 10 min (40 Hz, 80 W) with an ultrasonicator DL-60D (Shanghaihengxin, Shanghai, China). After sonication explants were added to pre-sterilized flasks containing bacterial suspension and flasks were shaken gently at 25 rpm for 30 min at room temperature. Explants were then blot-dried with sterile paper towels and transferred onto Petri dishes containing 1/2 MS medium with 100 µM acetosyringone. The co-cultivation period was 1–3 days in the dark at 28°C.

The bacterial suspension was prepared from a single colony of *A. tumefaciens* strain EHA105 with pCAMBIA2300::p35s-G10H-nos::p35s-ORCA3-nos or pCAMBIA2300::p35s-ORCA3-nos, as well as pCAMBIA2300. Bacteria were incubated in liquid Luria Bertani (LB) medium containing 100 mg L^−1^ kanamycin and 100 mg L^−1^ rifampicin (Sigma, St Louis, MO, U.S.A.) and grown at 28°C for 36 h with shaking (150 rpm). The initial culture was diluted 1∶1000 with liquid LB medium and grown on a shaker (250 rpm) until the OD_600_ reached 0.8. Then the cells were centrifuged (2,000×g, 10 min) and the supernatant was removed. Bacteria were resuspended in liquid MS medium containing100 µM acetosyringone and the OD_600_ was adjusted to 0.5. Finally, the bacteria were shaken (100 rpm) for another 2 h at room temperature.

After co-cultivation, the explants were transferred to callus induction medium containing 1.0 mg L^−1^ 2,4-Dichlorophenoxyacetic acid (2, 4-D), 1.0 mg L^−1^ 1-Naphthaleneacetic acid (NAA), 0.1 mg L^−1^ zeatin, 250 mg L^−1^ carbenicillin and 40 mg L^−1^ kanamycin for 10 days. Then the explants were transferred to the medium supplemented with 5.0 mg L^−1^ 6-benzylaminoputine (BA), 0.5 mg L^−1^ NAA, 250 mg L^−1^ carbenicillin and 70 mg L^−1^ kanamycin, which was effective on shoot initiation for 10 days. The resulting explants were transferred to shoot elongation medium supplemented with 1.75 mg L^−1^ BA, 0.55 mg L^−1^ Indole-3-acetic acid (IAA), 250 mg L^−1^ carbenicillin and 90 mg L^−1^ kanamycin, and subcultured every week. After three weeks, the elongated shoots were separated and transferred to root initiation medium (1/2 MS) containing 500 mg L^−1^ carbenicillin and 90 mg L^−1^ kanamycin for one month. Rooted plantlets were placed into sterile pot mixtures in a mist chamber for one month before transferred to normal greenhouse. The culture conditions were performed as described above for the plant seeding. All medium used in the study for *C. roseus* regeneration was based on Murashige and Skoog (MS) medium supplemented with 150 mg L^−1^ casein hydrolysate, 250 mg L^−1^ L-proline, 30 g L^−1^ sucrose, and 3 g L^−1^ gelrite. The pH of the medium was adjusted to 5.8 before autoclaving at 121°C for 22 min. Wild type plants were transformed with EHA105 containing pCAMBIA2300 and regenerated as the controls.

### PCR Analysis, Southern Hybridization and Real Time PCR

Genomic DNA was isolated from the young leaves of regenerated plants using CTAB method (Murray and Thompson 1980). Regenerated plantlets were screened by PCR using the forward primer 35s-F1 (5′-CGCACAATCCCACTATCCTT-3′), reverse primer ORCA3-R2 (5′-GCCCTTATACCGGTTCCAAT-3′) and G10H-R2 (5′-TGAATTCCTTGGCAGAATCC-3′) to detect the presence of target genes in the host. Positive plants were subjected to Southern blot analysis and real time PCR.

The integration of the ORCA3 and G10H gene in the transgenic *C. roseus* was investigated by Southern hybridization. Approxmately 80 µg of genomic DNA per sample was digested with *Hin*dIII and *Bgl*I, which were unique sites in the plasmid. The digested DNA was fractionated by 1.0%-agarose-gel electrophoresis, transferred on to a positively charged Hybond-N^+^ nylon membrane (GE Healthcare, USA) and hybridized with an alkaline-phosphatase-labeled partial DNA sequence of *p35s* as the probe. The probe (402 bp) was generated by PCR with primers 35s-F1 (5′-CGCACAATCCCACTATCCTT-3′) and ORCA3-R2 (5′-GCCCTTATACCGGTTCCAAT-3′). Hybridization and signal detection were performed using Amersham AlkPhos Direct Labeling Reagents (GE Healthcare, USA) and CDP-Star Detection Module following the manufacturer’s instructions. The hybridized signals were visualized by exposure to FujiX-ray film at room temperature (22°C for 12 h).

For real time PCR, total RNA was isolated from the leaves stored at −20°C. DNA contamination was removed using DNase I following the protocol provided by the manufacturer (TaKaRa, Japan). The cDNAs were synthesized from the RNA samples using Prime Script™ Reverse Transcriptase Reagent according to the manufacturer’s instructions, using oligo (dT) as the primer. The qRT-PCR analysis was performed in a Peltier Thermal Cycler PTC200 (Bio-Rad), using the cDNAs as a template and gene-specific primers for analysis. The primers for these genes (*ORCA3, AS, DXS, STR, TDC, G10H, D4H, CRMYC2 and RSP9*) are listed in Table S2 (from ExPlant Technologies B.V.). Ribosomal protein subunit 9 (Rsp9) was used as an internal control to evaluate all *C. roseus* plants. SYBR Green (SYBR Premix Ex Taq; TaKaRa) was used in the PCR reactions to quantify the amount of dsDNA. The relative Ct (threshold cycle value) method (User Bulletin 2, ABIPRISM700 Sequence Detection System, update 2001; PerkinElmer/Applied Biosystems) was used to estimate the initial amount of template present in the reactions.

### HPLC Analysis

Samples were prepared following the previously reported method [56]. Thirty mg dried leaves were used for each sample. The chromatography was carried out using an Agilent Eclipse XDB-C18 column (5 µm, 4.6×250 mm) (Phenomenex, Torrence, CA, USA). The chromatographic system was an Agilent Technologies 1200 series consisting of a G1322A Vacuum Degasser, a G1310A Iso Pump, a G1329A AutoSampler, a G1316A Thermostated Column Compartment and a G1315D Diode Array Detector.

The chromatographic method was developed for the qualitative and quantitative analysis of a variety of MIA and precursors. The mobile phase consisted of a mixture of 5 mM Na_2_HPO_4_ (pH adjusted to 6 with HCL) (solvent A) and methanol (solvent B) at a flow rate of 1.5 mL per min. The eluent profile (volume of solvent A/volume of solvent B) was: 0–2 min, linear gradient from 86∶14 to 14∶86; 26–30 min, isocratic elution with 14∶86 (v/v); 30–35 min, linear gradient from 14∶86 to 86∶14; 35–37 min, isocratic elution with 86∶14(v/v). The injected volume was 30 µL. Detection was with UV at 210–400 nm.

A mixture of reference standards of MIA and precursors (strictosidine and secologanin were from Phytoconsult, Leiden, The Netherlands; loganic acid, loganin, tabersonine, and vindoline were bought from PhytoLab, Vestenbergsgreuth, Germany; tryptamine was purchased from Aldrich Chemical, Milwaukee, WIS, USA; tryptophan and ajmalicine were purchased from Sigma-Aldrich, St. Louis, MO, USA; serpentine was purchased from Roth, Karlsruhe, Germany; catharanthine, anhydrovinblastine, vinblastine, and vincristine were kind gifts from Pierre Fabre, Gaillac, France) were detected and identified (Fig. S6 and Fig. S7) based on UV analysis of absorbance chromatograms [57]. Samples were applied in triplicate for quantification using calibration curves of the standards.

### NMR Analysis

After the GO and control plants were transplanted into the field for 6 months, their fresh leaves were harvested and grinded into powder with liquid N_2_, and then dried by frozen drier for two days. 50 mg of dried leaves was transferred to a 2 mL eppendorf tube to which 1.5 mL of CD_3_OD (750 µL) and D_2_O (750 µL) (KH_2_PO_4_ buffer, pH 6.0), containing 0.01% TSP, were added. The mixture was vortexed for 1 min, ultrasonicated for 30 min, and centrifuged for 20 min at 13000 rpm at room temperature. 800 µL of the supernatant from the mixture was transferred to a new eppendorf tube for a second centrifugation at 13,000 rpm for 5 minutes. 700 µL of the supernatant was transferred to a 5 mm NMR tube and used for the ^1^H-NMR analysis. Both CD_3_OD and D_2_O were purchased from Cambridge Isotope Laboratories, Inc., Andover, MA, USA.

All spectra were recorded at 25°C on a 500 MHz Bruker DMX-600 spectrometer (Bruker, Karlsruhe, Germany) operating at a proton NMR frequency of 500 MHz. For each sample, 128 scans were recorded with the following parameters: 0.126 Hz/point, pulse width (PW)30° (4.0 µs), and relaxation delay (RD)1.5 s. Free induction decays (FIDs) were Fourier transformed with line broadening factor = 0.3 Hz. The resulting spectra were manually phased and baseline corrected, and calibrated to TMSP at 0.0 ppm, using Topspin (version 2.1, Bruker).

The ^1^H-NMR spectra from direct extraction were automatically reduced to ASCII files by AMIX (version 3.7, Bruker). Spectral intensities were scaled to an internal standard and reduced to integrated regions of equal width (0.04) corresponding to the region of δ 0.0–10.0. The regions of δ 4.64–4.96 and δ 3.32 were excluded from the analysis because of the residual signal of D_2_O and CD_3_OD, respectively. Principal component analysis (PCA) and partial least squares discriminant analysis (PLS-DA) were performed with the SIMCA-P software (version 11.0, Umetrics, Umeå, Sweden). For scaling, the Pareto and unit variance methods were used for PCA and PLS-DA, respectively.

### Data Statistical Analysis

All experiments were conducted with three replicates. Statistical analysis was performed using one way analysis of variance (ANOVA) followed by Duncan’s Multiple Range (DMRT) test. The values are mean±SD for three samples in each group. *p* values ≤0.05 were considered as significant. The ANOVA for all the data was performed by SPSS (version 14.0, Chicago, IL, USA).

## Supporting Information

Figure S1 Scheme of construct of vectors with restriction sites. A: vector of pCAMBIA2300:: p35s-ORCA3-nos was constructed to overexpress ORCA3; B: vector of pCAMBIA2300::p35s-G10H-nos::p35s-ORCA3-nos was constructed to co-overexpress ORCA3 and G10H.
(TIF)

Figure S2 Southern blot of OR43 and GO84 plants. The fragment of p35s was used as the probe. +: pGO plasmid as the positive control; M: λ-HindIII Marker.
(TIF)

Figure S3 Regeneration and transformation of *C. roseus*. A: Callus induced from hypocotyls; B: plantlets with shoots and roots; C: Shoot initiation from callus; D: Transgenic plantlets in soil; E: Transgenic and control plants in soil before flowering, six plants at right side were transgenic lines, six at left side were control plants; F: Transgenic and control plants in soil after flowering, three plants at right side were transgenic lines, three at left side were control plants.
(TIF)

Figure S4 The levels of several important TIA in OR plants, GO plants and wild type *C. roseus* plants (the value of CK is the average of 15 wild type plants). “*”: significant increase (p<0.05 by ANOVA)
(TIF)

Figure S5 PCA score plot (A) and validate model of PLS-DA (B) for GO and control lines.
(TIF)

Figure S6 A: HPLC spectrum of the mixture of reference compounds; B: HPLC spectrum of one transgenic sample. 1: vindoline; 2: vincristine; 3: catharanthine; 4: vinblastine; 5: ajmalicine; 6: anhydrovinblastine; 7: tabersonine.
(TIF)

Figure S7 UV absorbance chromatograms of vindoline, catharanthine, ajmalicine, anhydrovinblastine and vinblastine.
(TIF)

Table S1 ^1^H NMR chemical shifts (δ) and coupling constants (Hz) of identified metabolites based on ^1^H-NMR, *J*-resolve, COSY, HSQC and references
(DOC)

Table S2 Primers list for Real Time PCR.
(DOC)
