# Supplementary figures and images for: Overexpression of ORCA3 and G10H in Catharanthus roseus Plants Regulated Alkaloid Biosynthesis and Metabolism Revealed by NMR-Metabolomics
Source: PLoS One. 2012 Aug 20;7(8):e43038. doi: 10.1371/journal.pone.0043038 (PMC3423439; doi:10.1371/journal.pone.0043038)

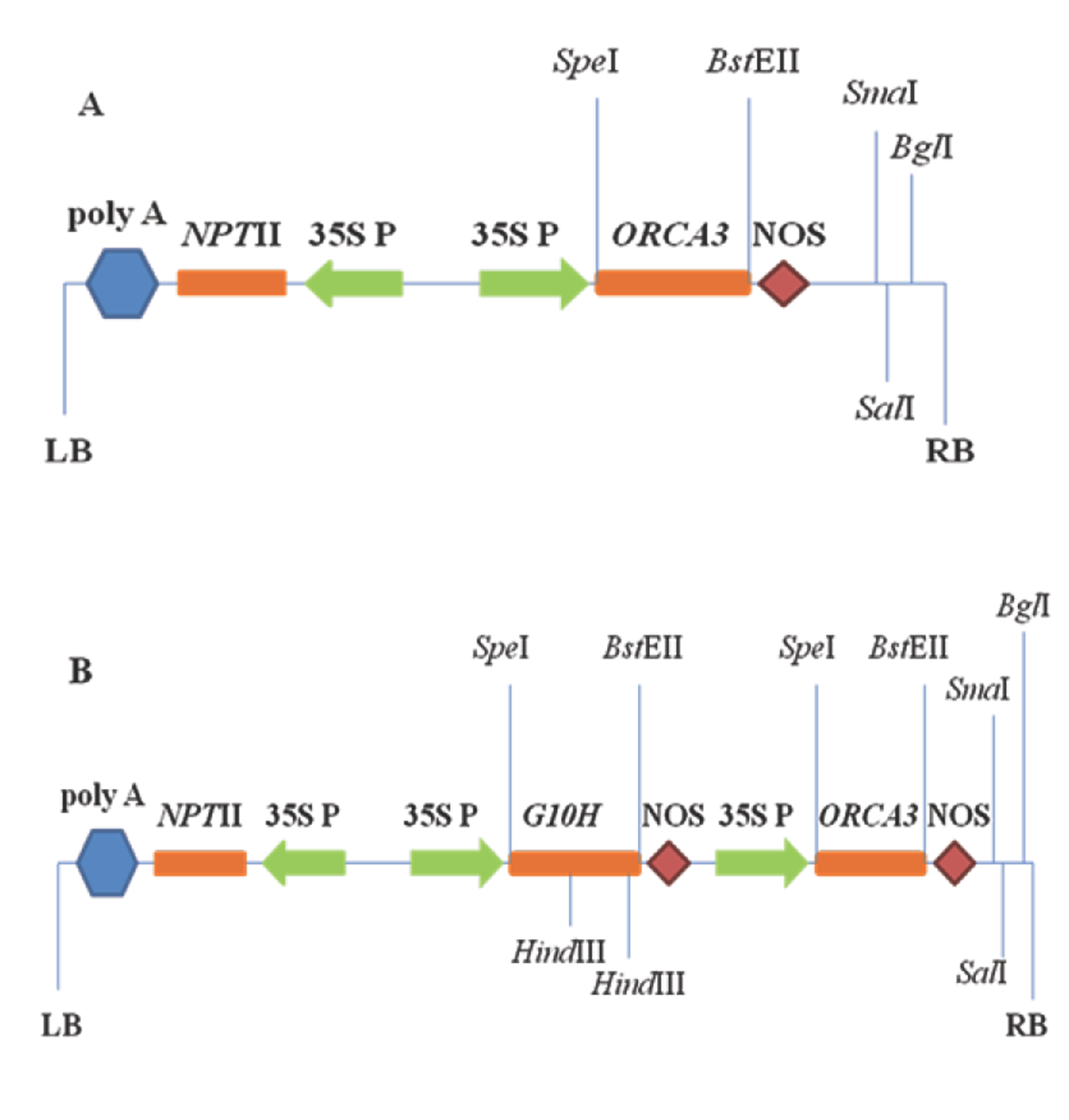

Supplement: Figure S1 — Scheme of construct of vectors with restriction sites. A: vector of pCAMBIA2300:: p35s-ORCA3-nos was constructed to overexpress ORCA3; B: vector of pCAMBIA2300::p35s-G10H-nos::p35s-ORCA3-nos was constructed to co-overexpress ORCA3 and G10H. (TIF) [file pone.0043038.s001.tif]

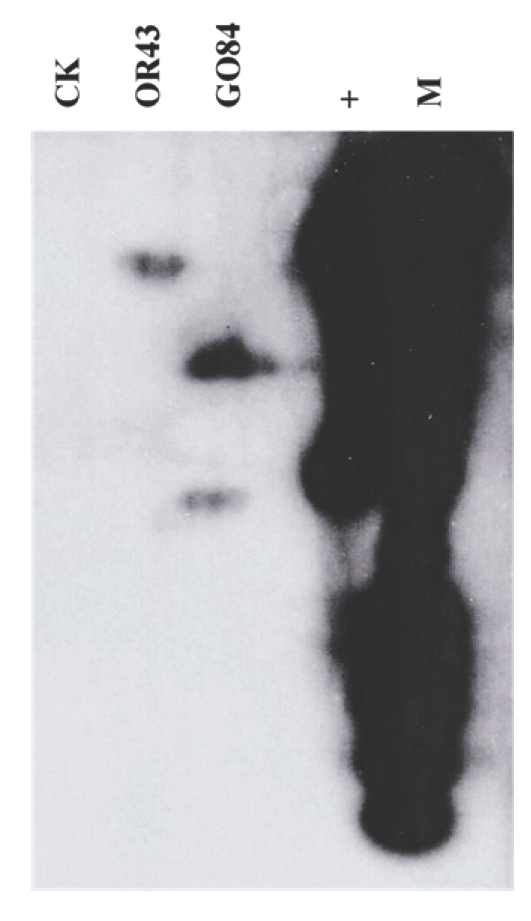

Supplement: Figure S2 — Southern blot of OR43 and GO84 plants. The fragment of p35s was used as the probe. +: pGO plasmid as the positive control; M: λ-HindIII Marker. (TIF) [file pone.0043038.s002.tif]

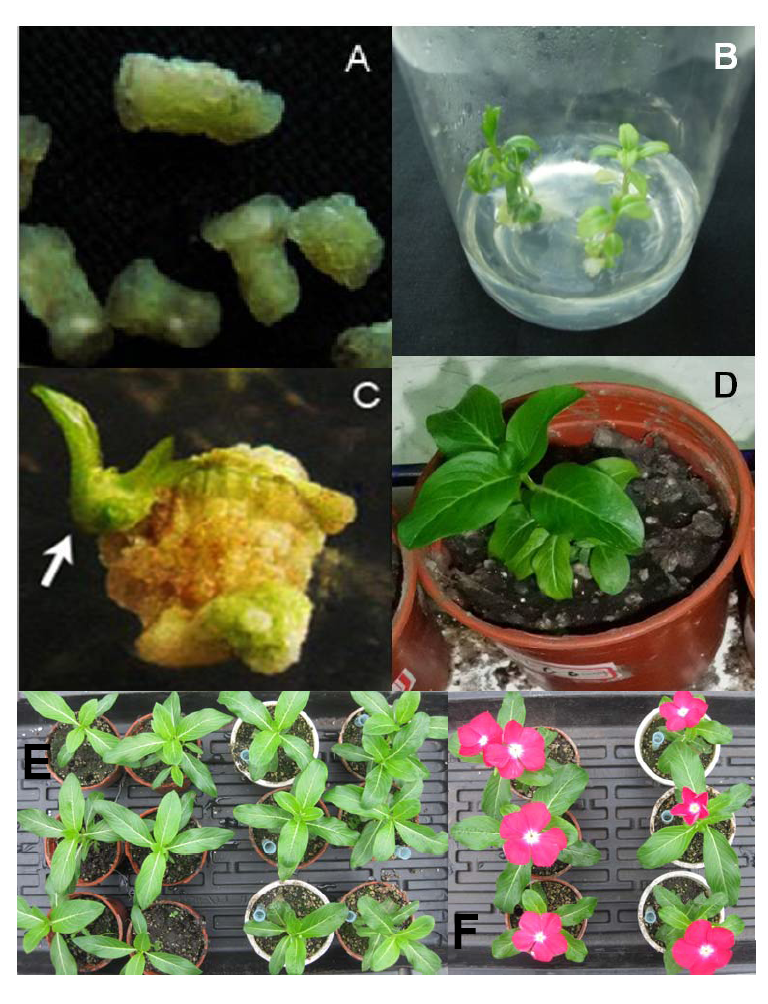

Supplement: Figure S3 — Regeneration and transformation of C. roseus. A: Callus induced from hypocotyls; B: plantlets with shoots and roots; C: Shoot initiation from callus; D: Transgenic plantlets in soil; E: Transgenic and control plants in soil before flowering, six plants at right side were transgenic lines, six at left side were control plants; F: Transgenic and control plants in soil after flowering, three plants at right side were transgenic lines, three at left side were control plants. (TIF) [file pone.0043038.s003.tif]

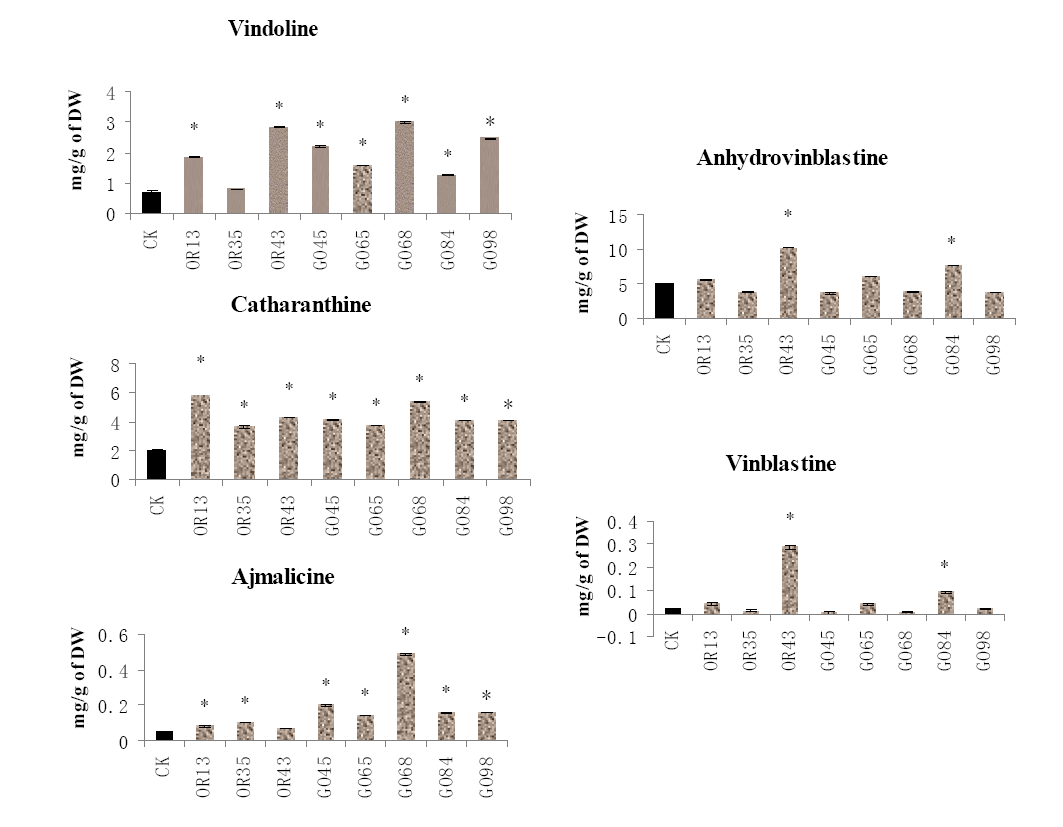

Supplement: Figure S4 — The levels of several important TIA in OR plants, GO plants and wild type C. roseus plants (the value of CK is the average of 15 wild type plants). “*”: significant increase (p<0.05 by ANOVA) (TIF) [file pone.0043038.s004.tif]

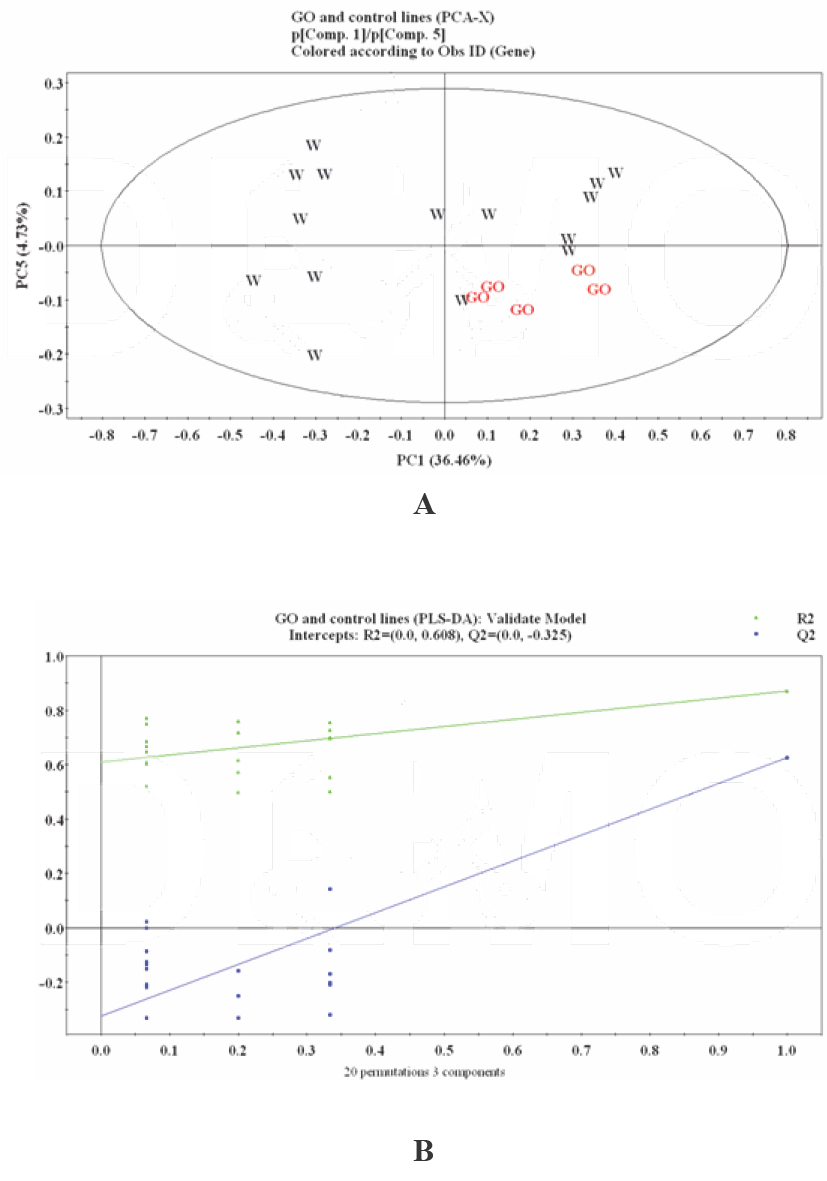

Supplement: Figure S5 — PCA score plot (A) and validate model of PLS-DA (B) for GO and control lines. (TIF) [file pone.0043038.s005.tif]

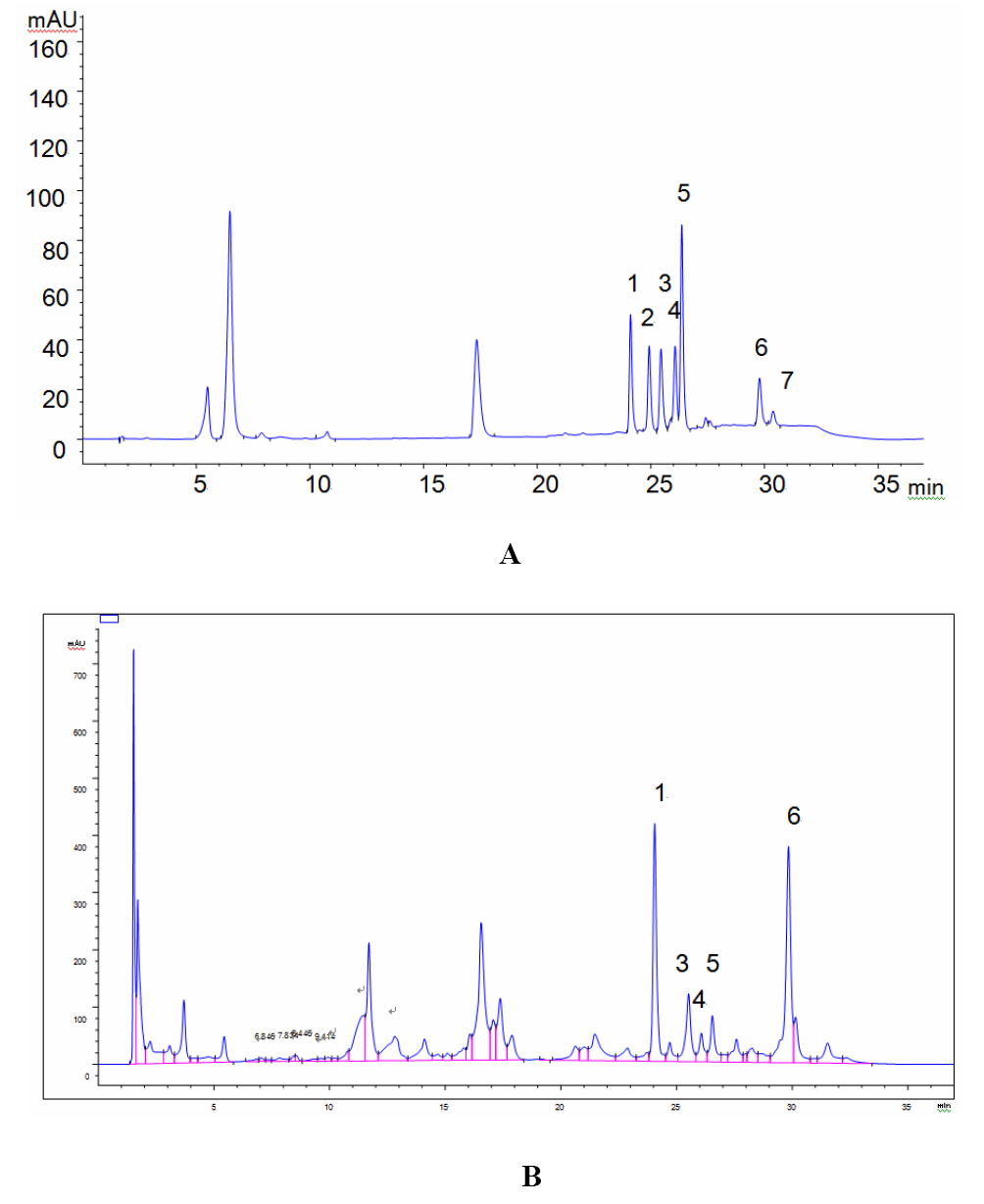

Supplement: Figure S6 — A: HPLC spectrum of the mixture of reference compounds; B: HPLC spectrum of one transgenic sample. 1: vindoline; 2: vincristine; 3: catharanthine; 4: vinblastine; 5: ajmalicine; 6: anhydrovinblastine; 7: tabersonine. (TIF) [file pone.0043038.s006.tif]

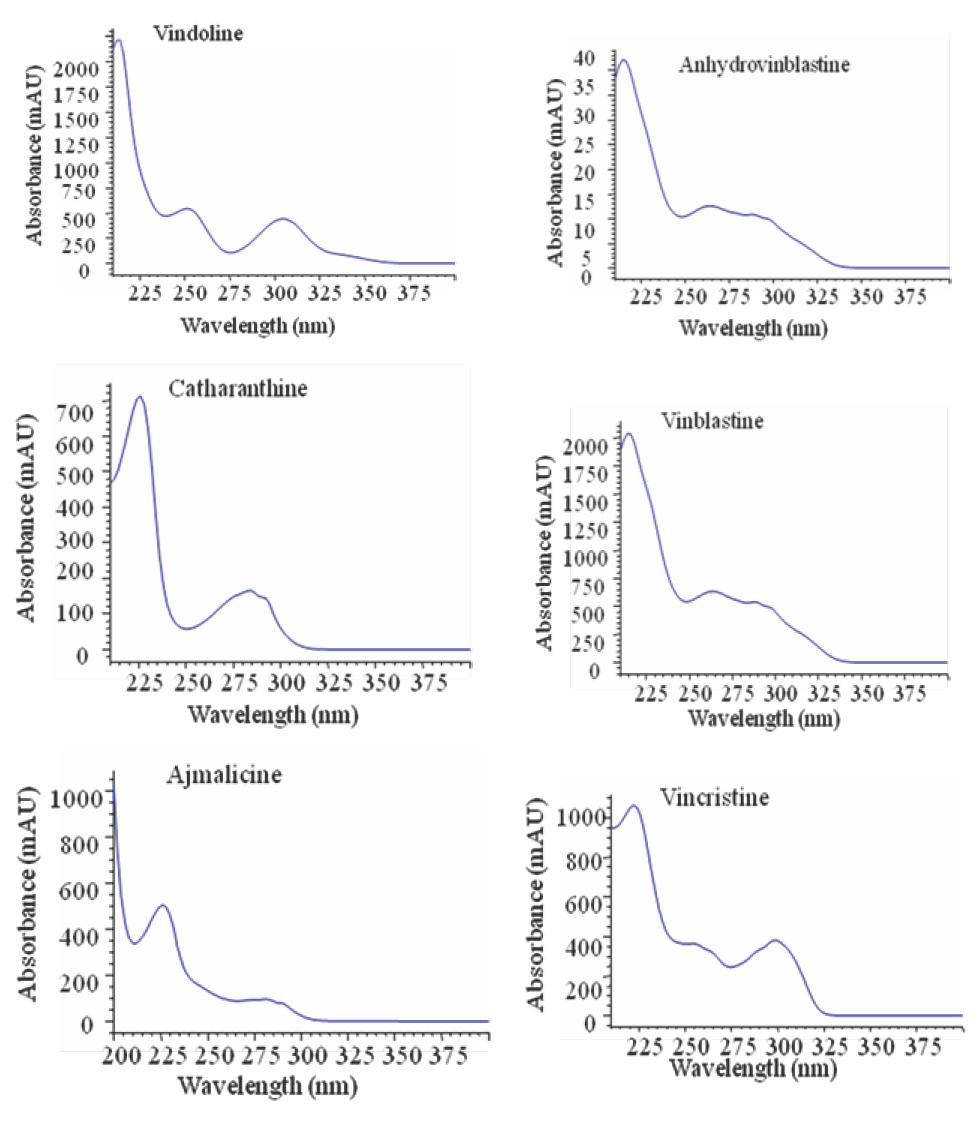

Supplement: Figure S7 — UV absorbance chromatograms of vindoline, catharanthine, ajmalicine, anhydrovinblastine and vinblastine. (TIF) [file pone.0043038.s007.tif]
