# Supplementary material for: Overexpression of ORCA3 and G10H in Catharanthus roseus Plants Regulated Alkaloid Biosynthesis and Metabolism Revealed by NMR-Metabolomics
Source: PLoS One. 2012 Aug 20;7(8):e43038. doi: 10.1371/journal.pone.0043038 (PMC3423439; doi:10.1371/journal.pone.0043038)
Supplement: Table S1 — 1H NMR chemical shifts (δ) and coupling constants (Hz) of identified metabolites based on 1H-NMR, J-resolve, COSY, HSQC and references (DOC) [file pone.0043038.s008.doc]

Table S1. ^1^H NMR chemical shifts (δ) and coupling constants (Hz) of identified metabolites based on ^1^H-NMR, *J*-resolve, COSY, HSQC and references

| **Compounds** | **Chemical shifts (δ)** |
| --- | --- |
| Isoleucine | 0.96 (t, J = 7.4), 1.03 (d, J = 6.8) |
| Leucine | 0.97 (d, J = 6.3), 0.99 (d, J = 6.3) |
| Valine | 1.01 (d, J = 7.0), 1.06 (d, J = 7.0), 2.28 (m) |
| Threonine | 1.34 (d, J = 6.6) |
| Alanine | 1.48 (d, J = 7.2) |
| Arginine | 1.70 (m), 1.90 (m) |
| Glutamic acid | 2.04 (m), 2.12 (m), 2.39 (m) |
| Glutamine | 2.15 (m), 2.48 (m) |
| Aspartic acid | 2.64 (dd), 3.83 (dd) |
| Asparagine | 2.82 (dd, J = 16.9, 8.2), 2.96 (dd, J = 16.9, 3.9) |
| Serine | 3.78 (dd, J = 6.2, 3.7), 3.92 (dd, J = 12, 6.2), 3.98 (dd, J = 12, 3.7) |
| 2,3-butanediol | 1.15 (d, J = 6.4), 3.50 (m) |
| EtOH | 1.19 (t, J = 7.1) |
| Quinic acid | 1.88 (dd), 1.93 (m) |
| Lactic acid | 1.37 (d, J = 7.2) |
| Acetic acid | 1.94 (s) |
| Malic acid | 2.45 (dd, J = 15.6, 7.2), 2.72 (dd, J = 15.6, 3.9) |
| Citric acid | 2.44 (d, J = 15.6), 2.71 (d, J = 15.6) |
| Ketoglutaric acid | 3.00 (t, J = 7.5) |
| Succinic acid | 2.51 (s) |
| Oxalacetic acid | 3.65 (s) |
| Fumaric acid | 6.55 (s) |
| Sucrose | 5.41 (d, J = 3.8) |
| α-glucose | 5.18 (d, J = 3.7) |
| β-glucose | 4.58 (d, J = 7.9) |
| Choline | 3.21 (s) |
| Chlorogenic acid | 7.61 (d, *J* = 15.9), 7.14 (d, *J* = 2.1), 7.05 (dd, *J* = 8.4, 6.36 (d, *J* = 15.9) |
| 4-O-Caffeoyl quinic acid | 7.67 (d, *J* = 15.9), 7.17 (d, *J* = 2.1), 7.08 (dd, *J* = 8.3, 2.0), 2.1), 6.44 (d, *J* = 15.9), 2.09 (m) |
| 2,3-DHBA | 6.83 (t, J = 8.0), 7.26 (dd, J = 8.1, 1.5), 7.52 (dd, J = 7.9, 1.5) |
| Quercetin-3-O-glucoside | 7.87 (d, J = 2.1), 7.65 (dd, J = 8.5, 2.1), 6.99 (d, J = 9.3), 6.31 (d, J = 2.1), 6.50 (d, J = 2.1), 5.33 (d, J = 6.8) |
| Kaempferol 1 | 6.30 (d, J = 2.1), 6.51 (d, J = 2.1), 7.00 (d, J = 9.3), 8.04 (dd, J = 9.0, 2.2) |
| Kaempferol 2 | 6.30 (d, J = 2.1), 6.51 (d, J = 2.1), 6.98 (d, J = 9.3), 8.08 (dd, J = 8.9, 1.4) |
| Loganic acid | 7.06 (d, *J* = 1.1), 5.30 (d, *J* = 3.2), 4.72 (d, *J* = 8.0), 1.07 (d, *J* = 6.9). |
| Secologanin | 7.44 (dd, J = 11.7, 0.9), 7.56 (d, J = 1.9), 9.65 (d, J = 1.4) |
| Catharanthine | 1.10 (t, J = 7.3), 7.36 (d, J = 8), 7.55 (d, J = 8) |
| Strictosidine | 7.80 (s) |
| Serpentine | 8.31 (d), 8.35 (d), 8.46 (d) |
| Vindoline | 0.51 (t, J = 7.4), 2.00 (s), 2.66 (s), 5.93 (m), 6.22 (d, J = 2.3), 7.11 (d, J = 8) |
| Vindolinine | 1.08 (d, J = 5.6), 5.92 (m), 6.51 (dd, J = 9.9, 3.1), 6.82 (d, J = 7.9), 7.24 (d, J = 7.3) |
