# Supplementary material for: Overexpression of ORCA3 and G10H in Catharanthus roseus Plants Regulated Alkaloid Biosynthesis and Metabolism Revealed by NMR-Metabolomics
Source: PLoS One. 2012 Aug 20;7(8):e43038. doi: 10.1371/journal.pone.0043038 (PMC3423439; doi:10.1371/journal.pone.0043038)
Supplement: Table S2 — Primers list for Real Time PCR. (DOC) [file pone.0043038.s009.doc]

Table S2 Primers list for Real Time PCR.

| Gene | Accession no | Primer pairs | Product(bp) |
| --- | --- | --- | --- |
| ORCA3 | AJ251249 | 5’-CCGGACCCGTTAGAGTAAACC-3’ | 112 |
|  |  | 5’-CGTCTCTTCTTCCTTCCTCCAC-3’ |  |
| G10H | AJ251269 | 5'-GGTAGCCTCACGATGGAGAA-3' | 247 |
|  |  | 5'-CCTTGGCAGAATCCGAATAA-3' |  |
| AS | AF441857 | 5’-GCGAACATTTGCAGATCCAT-3’ | 156 |
|  |  | 5’-GGCCGATTTGTTATTGTTCC-3’ |  |
| DXS | AJ011840 | 5’-TCGCTGCAGAACTTAGAGCA-3’ | 146 |
|  |  | 5’-GCCAACATCCCAAATGATTC-3’ |  |
| TDC | X67662 | 5'-CGCCTGTATATGTCCCGAGT-3' | 227 |
|  |  | 5'-GTTGCGATTTGCCAATTTTT-3' |  |
| STR | X61932 | 5'-TGCCACACAACTAGCCACAA-3' | 146 |
|  |  | 5'-TCATGATTTCTTCCACACCTTCG-3' |  |
| D4H | AF008597 | 5’-TACCCTGCATGCCCTCAACCA-3’ | 151 |
|  |  | 5’-AGAGCTCCAGGAATGAAGGGG-3’ |  |
| CrMYC2 | AF283507 | 5'-TTTGGCAGTCGTCTGTTGTC-3' | 226 |
|  |  | 5'-TCGGTATCGGTCACCTCTTC-3' |  |
| Rsp9 | AJ749993 | 5'-TCCACCATGCCAGAGTGCTCATTAGG-3' | 190 |
|  |  | 5'-TCCATCACCACCAGATGCCTTCTTCG-3' |  |
